# Tuning structure and mechanical properties of Ta-C coatings by N-alloying and vacancy population

**DOI:** 10.1038/s41598-018-35870-x

**Published:** 2018-12-05

**Authors:** T. Glechner, P. H. Mayrhofer, D. Holec, S. Fritze, E. Lewin, V. Paneta, D. Primetzhofer, S. Kolozsvári, H. Riedl

**Affiliations:** 1 Institute of Materials Science and Technology, TU Wien, A-1060 Wien, Austria; 2 Department of Materials Science, Montanuniversität Leoben, A-8700 Leoben, Austria; 3 Department of Chemistry - Ångström Laboratory, Uppsala University, SE-75120 Uppsala, Sweden; 4 Department of Physics and Astronomy, Uppsala University, SE-75120 Uppsala, Sweden; 5 Plansee Composite Materials GmbH, D-86983 Lechbruck am See, Germany

## Abstract

Tailoring mechanical properties of transition metal carbides by substituting carbon with nitrogen atoms is a highly interesting approach, as thereby the bonding state changes towards a more metallic like character and thus ductility can be increased. Based on ab initio calculations we could prove experimentally, that up to a nitrogen content of about 68% on the non-metallic sublattice, Ta-C-N crystals prevail a face centered cubic structure for sputter deposited thin films. The cubic structure is partly stabilized by non-metallic as well as Ta vacancies – the latter are decisive for nitrogen rich compositions. With increasing nitrogen content, the originally super-hard fcc-TaC_0.71_ thin films soften from 40 GPa to 26 GPa for TaC_0.33_N_0.67_, accompanied by a decrease of the indentation modulus. With increasing nitrogen on the non-metallic sublattice (hence, decreasing C) the damage tolerance of Ta-C based coatings increases, when characterized after the Pugh and Pettifor criteria. Consequently, varying the non-metallic sublattice population allows for an effective tuning and designing of intrinsic coating properties.

## Introduction

Transition metal (TM) carbides and nitrides belong to the class of ultra-high temperature ceramics (UHTC) exhibiting highly interesting properties. Various members of this materials family present a unique mix of high hardness^1^, high thermal and chemical stability^2^, accompanied by electrical resistivity that is comparable to their pure transition metals^3^ (e.g. Ta-C 25 µΩ cm)^4^. Therefore, these TM ceramics are well established in various industrial applications, especially in form of thin films. Here, the field of protective coatings but also microelectronics (e.g. diffusion barriers or piezo-electric applications) gained a lot of scientific and industrial attention for nitride-based systems containing Ti and/or Al^5^. Among the TM carbides, Ta-C prevail the highest melting temperature of all known binary systems (3983 °C)^6^ and in relation to the predominant covalent-metallic bonding character extreme hardness and chemical inertness^1,7^. However, for a broad utilization of Ta-C based materials – either as structural components or thin films – the tremendously low ductility and extremely high transition temperature, where macroscopic ductile behavior is observed (about 0.5·T_m_)^8^, are major limiting issues. In general, improving the ductility of TM carbides (as well as nitrides) while retaining other thermo-mechanical properties is highly desired for a variety of applications. Basic mechanisms to increase the ductility of ceramic materials are the incorporation of ductile phases, grain boundary engineering, or varying crystallite and grain sizes^9–11^. For thin film materials, architectural arrangements such as superlattices or hierarchical composites achieved interesting results concerning fracture toughness enhancements^12,13^ – K_IC_ increases by an order of magnitude. However, for an intrinsic increase of the ductility, thin film materials design needs to be conducted on the atomic scale range, adapting the bonding character^14^. Ductility and toughness, e.g. fracture toughness descriptors such as K_IC_, are indirect connected, as ductility is the plastic strain to fracture whereas fracture toughness is a parameter based on fracture mechanics originating from the adsorbed energy to fracture. Nevertheless, to intrinsically reduce brittle behavior of thin film materials the prevalent bonding character is of major interest. Several studies have considered alloying on the metallic sublattice and hence tailoring the electronic structure^15,16^. For examples, Kindlund *et al*. showed that adding Mo to VN to form V_0.5_Mo_0.5_N increases ductility through tuning the occupancy of d–t_2g_ metallic bonding states^17^. Furthermore, also vacancies have been shown to enhance ductility of V_0.5_Mo_0.5_N^18^.

In this study we introduce an alternative approach: alloying on the non-metallic sublattice instead of the metallic one through a partial substitution of carbon by nitrogen in Ta-C. This method aims for solid-solutions of non-metallic atoms on their sublattice. Due to competing crystal structures of Ta-C and Ta-N at room temperature, a detailed understanding of the phase evolution for the ternary TaC_1−x_N_x_ system is of major importance for a knowledge-based targeted design of materials with enhanced fracture tolerance. In contrast to various TM carbides (e.g. Ta-C, Hf-C, Zr-C), which crystallize in the face centered cubic (fcc) NaCl (Fm$\bar{3}\,$m, #225) structure, Ta-N crystalizes predominantly in hexagonal (hex) structure types. Two highly similar prototypes, ε-TaN (P6/mmm, #191) and π-TaN (P$\bar{6}$ 2m, #189) are known for Ta-N, with π-TaN being the most stable one^19^. In an equilibrium state, π-TaN transforms to fcc δ-TaN (Fm$\bar{3}\,$m, #225) above 1950 °C^20^, highlighting that fcc structured TaN can be accessed through higher entropy (e.g. by introducing vacancies, as high temperature phases are typically of higher symmetry but also increased disorder). This has recently been verified through detailed ab initio calculations, showing that depending on the vacancy type (metallic or non-metallic sublattice) and content, δ-TaN can be stabilized over π-TaN even at 0 K^21^. For Ta-C, non-metallic vacancies are more decisive than metal vacancies for promoting a stabilization of the cubic structure, whereas for Ta-N, metallic vacancies are of crucial importance^1,22^. In general, the vacancy stabilized structures may also influence the hardness, especially when thereby the valence electron concentrations (VEC) is tuned towards 8.4 e/f.u.^23^. For the individual TM-carbides, this leads to individual non-stoichiometric compositions for optimized hardness^23^. A ductile behavior of vacancy stabilized (cubic) Ta-N has recently been proposed by density functional theory (DFT) calculations^22^. Physical vapor deposition (PVD) is known to access metastable phases due to highly limited kinetics during film growth (extremely high cooling rates 10^13^ K·s^−1^) and a pronounced introduction of structural defects, such as point defects. Considering all these aspects, a stabilization of fcc structured Ta-C-N by PVD is a plausible scenario, and indeed has been already reported before^24^. To date, Ta-C-N thin films have only been employed as diffusion barriers between copper and silicon in semiconductor devices^25^. Their use as protective coating with excellent mechanical properties, especially in a highly crystalline constitution, is indeed relatively unexplored^24,26^.

In this work, we deployed a computational guided approach to give a profound structural and elastic characterization of the Ta-C-N system corroborated by experimental investigations on magnetron sputtered thin films using a Ta-C compound target in mixed Ar/N_2_ atmospheres. First, we discuss the phase formation with varying non-metal sublattice compositions of TaC_1−x_N_x_ − N/(C+N) ratio correlates to x – from the theoretical and experimental points of view. Subsequently, the mechanical properties with a special focus on guiding the experiments to obtain enhanced ductility are studied in detail. Since we use fcc structured Ta-C as the base-structure (also for our experiments), we avail nitrogen as an alloying element. Hence, for Ta-normalized chemical notations we use TaC_1−x_N_x_, but for general chemical information we will refer to our coatings as Ta_1−y−z_C_y_N_z_.

## Results and Discussion

**Figure 1:**
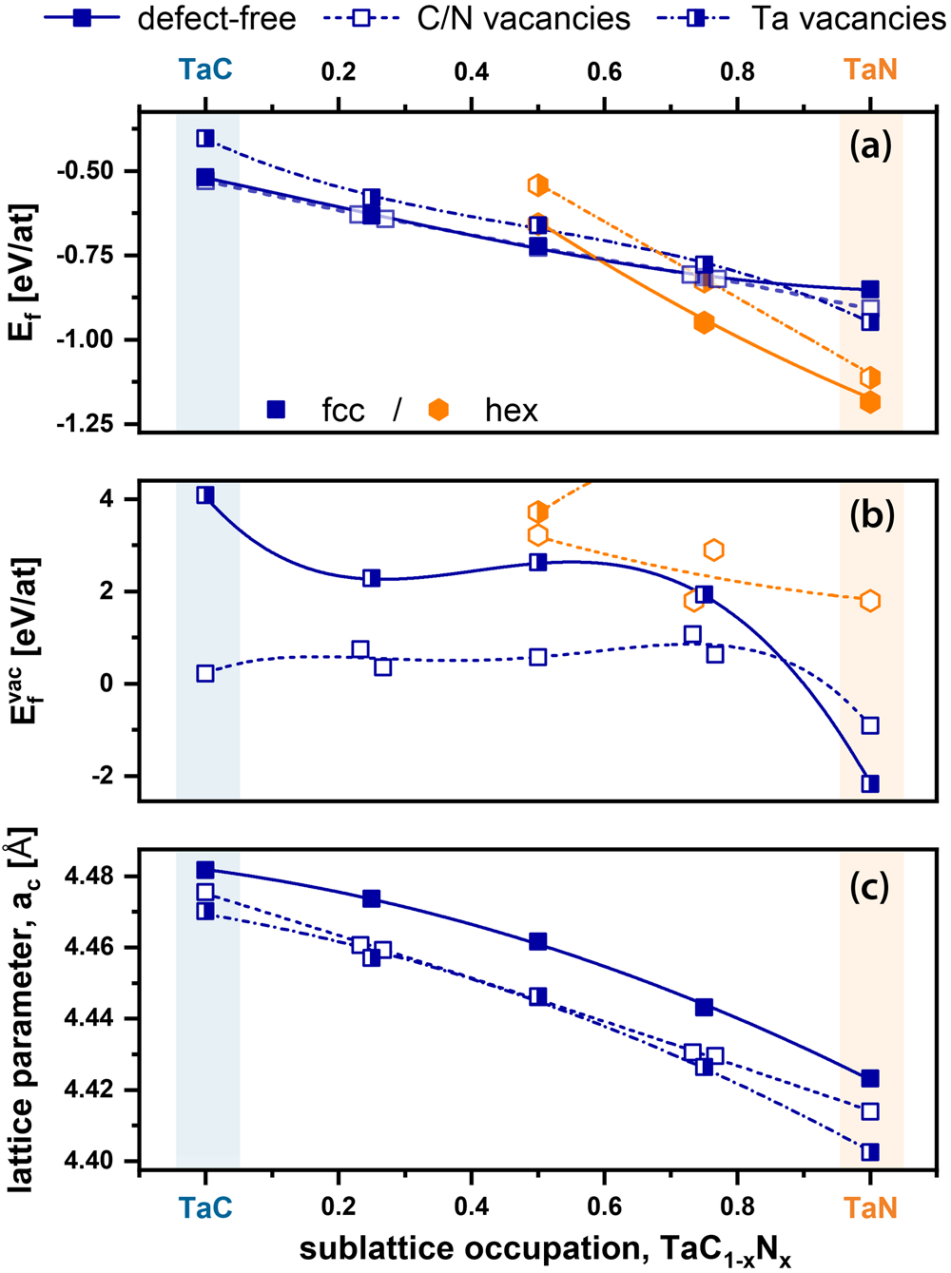
Phase transition, observing ab initio calculated E_f_ between fcc and hex structured TaC_1−x_N_x_ utilizing defect-free and metal deficient super cells (**a**). Energy of formation for specific vacancy types, ${E}_{f}^{vac}$, over the full compositional range (**b**). Calculated lattice parameters of defect-free and defected fcc structured TaC_1−x_N_x_ (**c**). Blue squares denote to fcc structured TaC_1−x_N_x_, whereas orange hexagons indicate hexagonal structures.

In order to theoretically explore the influence of nitrogen or carbon on the corresponding stable binary structures – fcc-TaC and hex-TaN – we varied the non-metallic sublattice occupation, x, of the TaC_1−x_N_x_ structures with increments of x = 0.25 in the DFT calculations. In Fig. 1a the energy of formation, E_f_, for defect-free and Ta deficient structures (two atoms removed on the Ta sublattice – vacancy content 6%) is presented – full symbols with solid lines denote the defect-free crystals, whereas half-filled symbols with dash-dotted lines denote to Ta vacant crystals. In the whole manuscript, blue squares refer to fcc crystals and orange hexagons to the hexagonal structure type. The energy of formation E_f_ was calculated using Eq. 1, where ${{\rm{E}}}_{0}^{{\rm{per}}/{\rm{def}}}$ denotes the total energy of the relaxed supercell that is divided by the total number of atoms in the cell. E_i_ is the total energy per atom of each element in its stable state at ambient conditions (bcc Ta, graphite C, and molecular N), and n_i_ is the number of atoms of each species.

1 $${{E}}_{{f}}={{E}}_{0}^{{per}/def}-\frac{1}{{\sum }_{i}{n}_{i}}\,\cdot \sum _{i}{n}_{i}{E}_{i}$$

In general, for the defect-free and Ta deficient cells, E_f_ decreases with increasing nitrogen content, and yields the transition between fcc and hex TaC_1−x_N_x_ in the range of x = 0.58. In accordance with earlier DFT studies by Koutná *et al*. (mentioning that the most stable configuration is Ta_0.78_N)^27^, we also observe a stabilization of fcc-TaN and highly nitrogen rich fcc-TaC_1−x_N_x_ through Ta vacancies (with respect to the perfect (defect-free) fcc-structures). The E_f_ difference between the defect-free and non-metallic vacant cells is only marginal and hence requires a further descriptor.

**Figure 2:**
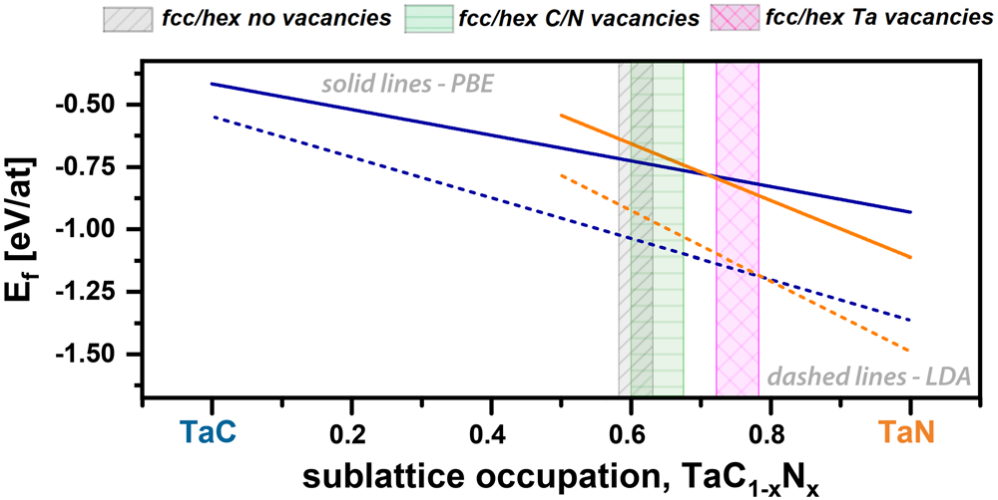
Phase intersection between fcc and hex TaC_1−x_N_x_ structure influenced by vacancies as well as different potentials utilized. The colored regions represent an error bar for this intersection, considering the different vacancy species types, obtained by PBE (left border) and LDA (right border) calculations. Solid and dashed lines show the E_f_ curve for the defect-free and Ta vacant (vacancy concentration of 6%) cells for PBE and LDA potential, respectively.

The stabilizing effects of vacancies on the individual sublattices (indicated here as Ta vacancies – by half-filled symbols - and non-metallic vacancies – by open symbols) are further quantified using the vacancy energy of formation, ${{\rm{E}}}_{{\rm{f}}}^{{\rm{vac}}}\,$calculated after^28^, as presented in Fig. 1b. The vacancy concentration is 6% for Ta vacancies as well as non-metallic (C and N) vacancies, which were randomly distributed. Here we clearly see, that for the fcc-structured cells the non-metallic vacancies are energetically more favorable than the Ta vacancies, whereas at high N contents (above x = 0.80), the formation energy of both metallic and non-metallic vacancies turns negative, indicating that their presence is necessary to stabilize the cubic phase (see blue open and half-filled squares in Fig. 1b). For the hexagonal structure type, we calculated ${{\rm{E}}}_{{\rm{f}}}^{{\rm{vac}}}$ only down to x = 0.5, as for all vacancy types ${{\rm{E}}}_{{\rm{f}}}^{{\rm{vac}}}$ is much higher as compared to the fcc cells. Due to the low ${{\rm{E}}}_{{\rm{f}}}^{{\rm{vac}}}$ of the non-metallic vacancies, 215 meV to 951 meV, nitrogen or carbon vacancies are promoted in fcc TaC_1−x_N_x_. To study whether N or C has a lower ${{\rm{E}}}_{{\rm{f}}}^{{\rm{vac}}}$ we performed selected calculations for 64-atom sized TaC_0.50_N_0.50_ and TaC_0.25_N_0.75_ supercells, from which we individually removed only one N or C atom. Thereby, we obtain that a carbon vacancy has a lower ${{\rm{E}}}_{{\rm{f}}}^{{\rm{vac}}}$ than a nitrogen vacancy for both compositions. Here we also want to mention that due to the finite supercell sizes a creation of vacancies leads to the change of short-range order parameters therefore the evaluation of the formation energy would require corrections as described in ref.^29^. There, the corrections are in the order of 0.2 eV for metallic vacancies in Ta_50_Al_0.5_N. Assuming that similar corrections would also apply to TaC_1−x_N_x_, the energy of formations may change by approximately 0.004 eV/at. Consequently, these corrections will not change the predicted phase stability trends qualitatively. To obtain a more assailable parameter for comparison with experiments, we calculated the lattice parameters for various fcc-TaC_1−x_N_x_ compositions, without and with 6% Ta or non-metallic vacancies, see Fig. 1c. Through the substitution of carbon by nitrogen, the lattice parameter decreases from around 4.48 Å for fcc-TaC to 4.43 Å for fcc-TaN. This agrees in principle with the atomic radii of C and N, C is slightly larger with r_C_ = 70 pm than N with r_N_ = 65 pm^30^. Tantalum as well as non-metallic vacancies decrease the lattice parameter, by about 0.02 Å/6 at.% vacancy concentration, over the full chemical composition range. These results also allow to monitor the accuracy of the DFT calculations by comparing the calculated lattice parameter of TaC and TaN with experimentally observed values. The experimentally obtained lattice parameter of fcc-TaC is reported with 4.45 Å^31^ (no indication of the stoichiometry) and in excellent agreement with our calculated ones of 4.42 Å and 4.48 Å using LDA and PBE exchange correlation potentials (xc), respectively. Especially for Ta-C, our former study^1^ highlights, that DFT (using PBE potentials) overestimates the lattice parameter and fcc Ta-C is compositionally stabilized by C-vacancies in the order of TaC_0.76_. The reported experimentally obtained lattice parameters for δ-TaN vary from 4.33 Å^32^ to 4.42 Å^33^, again in excellent agreement with our calculated lattice parameters of 4.36 Å (LDA) and 4.42 Å (PBE). The different potentials not only influence the lattice parameters, but also the chemical composition where the preferred phase changes from fcc to hex, see Fig. 2. When using PBE, the intersection of Ta-vacant fcc and hex E_f_ curves is at x = 0.72 (solid lines in Fig. 2), which shifts to x = 0.78 for LDA (dashed lines). This region (from x = 0.72 to 0.78) is marked with a purple hashed bar. The defect-free structures show a transition region between x = 0.62 (LDA) and 0.58 (PBE), indicated by a gray shaded striped bar. In contrast to Ta-vacancies, the non-metallic vacancies shift the phase transition to only slightly higher x-values - compared to the defect-free structure - between x = 0.68 (LDA) and 0.60 (PBE). Thus, the colored bars represent more or less an error bar of the intersection between fcc and hex TaC_1−x_N_x_. In summary, the DFT results pointed out that vacancies – both non-metallic and Ta ones – enable fcc TaC_1−x_N_x_ solid solutions up to higher nitrogen contents (as compared to defect-free structures).

**Figure 3:**
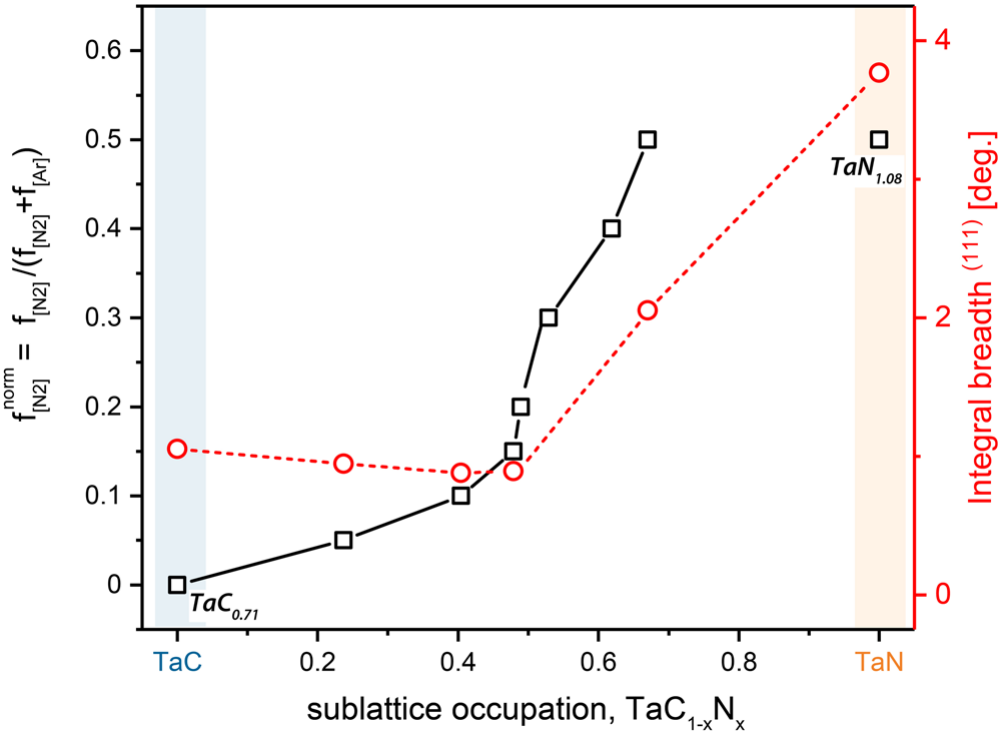
Relationship between the deposition conditions (nitrogen to total flow rate ${f}_{[{N}_{2}]}^{norm}$) and the experimentally determined N/(C + N) ratio, x (black squares), of the coatings. Integral breadth of the (111) peaks – see Fig. 4 – for corresponding ${f}_{[{N}_{2}]}^{norm}$ (red circles).

**Figure 4:**
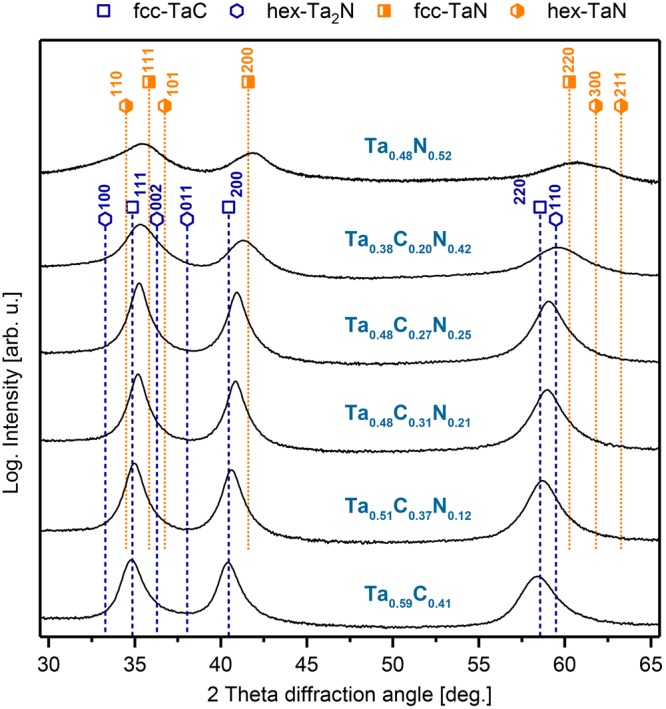
XRD patterns of powdered as deposited coatings with increasing nitrogen content. Reference lines: fcc-TaC^31^, fcc-TaN^32^, hex-Ta_2_C^34^, and hex-TaN^53^.

To corroborate the theoretically predicted stability trends, we subsequently performed phase and composition analysis of sputter-deposited Ta-C-N films. Figure 3 shows the relation between the used N_2_ flow rate ratio and chemical compositions of our coatings. Already a nitrogen flow rate ratio of only ${{\rm{f}}}_{[{{\rm{N}}}_{2}]}^{{\rm{norm}}}$ = 0.05 leads to a substitution of about 25% carbon atoms on the non-metallic sublattice with nitrogen, leading to a corresponding chemical composition of Ta_0.51_C_0.37_N_0.12_. This clear trend is obvious till ${{\rm{f}}}_{[{{\rm{N}}}_{2}]}^{{\rm{norm}}}$ = 0.15, replacing around half of the carbon atoms by nitrogen, Ta_0.48_C_0.27_N_0.25_. At even higher nitrogen flow rate ratios, e.g. ${{\rm{f}}}_{[{{\rm{N}}}_{2}]}^{{\rm{norm}}}$ = 0.3 or 0.5 (see details in Table 1), the overall metal to non-metal ratio decreases from around 1 to values between 0.75 or even 0.61. This is a strong deviation from the typical 1:1 ratio of metal and non-metal species in defect-free NaCl-type crystals, and needs to be correlated with structural investigations. Thus, any Ta-normalized notation of the type TaC_1−x_N_x_ would be misleading, as it hides the information on the metal/non-metal ratio. Therefore, the individual XRD patterns of Fig. 4 are indicated by the chemical compositions in the form of Ta_1−y−z_C_y_N_z_. The binary Ta_0.59_C_0.41_ coating (corresponding to TaC_0.71_) exhibits a dominant fcc structure, whereby the small right-hand shoulder of the 220 XRD peak may be due to a small fraction of the Ta_2_C phase^34^. Contrary, especially the C-rich ternary Ta_1−y-z_C_y_N_z_ compositions (up to Ta_0.48_C_0.27_N_0.25_) are single phase fcc structured. With increasing N content (due to the increased nitrogen flow rate ratio, see Fig. 3) the XRD peak positions shift from fcc-TaC towards fcc-TaN^31,32^. As mentioned above, the metal to non-metal ratio stays around 1, up to a nitrogen flow rate ratio of ${{\rm{f}}}_{[{{\rm{N}}}_{2}]}^{{\rm{norm}}}$ = 0.15, leading to this Ta_0.48_C_0.27_N_0.25_ film composition. The Ta_0.38_C_0.20_N_0.42_ coating, deposited at ${{\rm{f}}}_{[{{\rm{N}}}_{2}]}^{{\rm{norm}}}$ = 0.5, already exhibits rather broad XRD peaks (which are relatively close to the standard fcc-TaN positions), indicative for very small grain sizes. This is even more pronounced for the binary Ta_0.48_N_0.52_ coating (prepared with two Ta targets in N_2_ atmosphere), where the XRD investigations already suggest the co-existence of hex-TaN next to fcc-TaN. The competition of these two phases, leads to the extremely small grain sizes (as suggested by the rather broad XRD peaks). But the important point here is that TaN thermodynamically prefers to crystallize in stoichiometric hex structure with the N/Me ratio of 1. Thus, the N/Me ratio of 1.08 (Ta_0.48_N_0.52_) – which actually points towards Ta-vacancies – yields a tendency to stabilize the fcc structure. This is in excellent agreement with DFT predictions, which show that for Ta-vacant Ta-N compositions the fcc structure is more stable.

The calculated integral breadth of the 111 peaks, red open circles in Fig. 3, clearly supports the discussed structural changes. Up to the N_2_ flow rate ratio of 0.15, the integral breadth is almost constant at 0.8 deg. For higher N_2_ flow rate ratio the integral breadth significantly increases, suggesting the presence of additional phases (most likely hex) next to fcc-Ta_1−y−z_C_y_N_z_, leading to the rather small grain size. The observed phase transition from single-phase fcc-Ta_1−y−z_C_y_N_z_ to fcc plus hex Ta_1−y−z_C_y_N_z_ is at around x = 0.68 in the Ta-normalized notation of TaC_1−x_N_x_. This agrees with DFT calculations, especially when considering Ta vacancies, where the intersection is between x = 0.72 and 0.78 – see Fig. 2.

**Figure 5:**
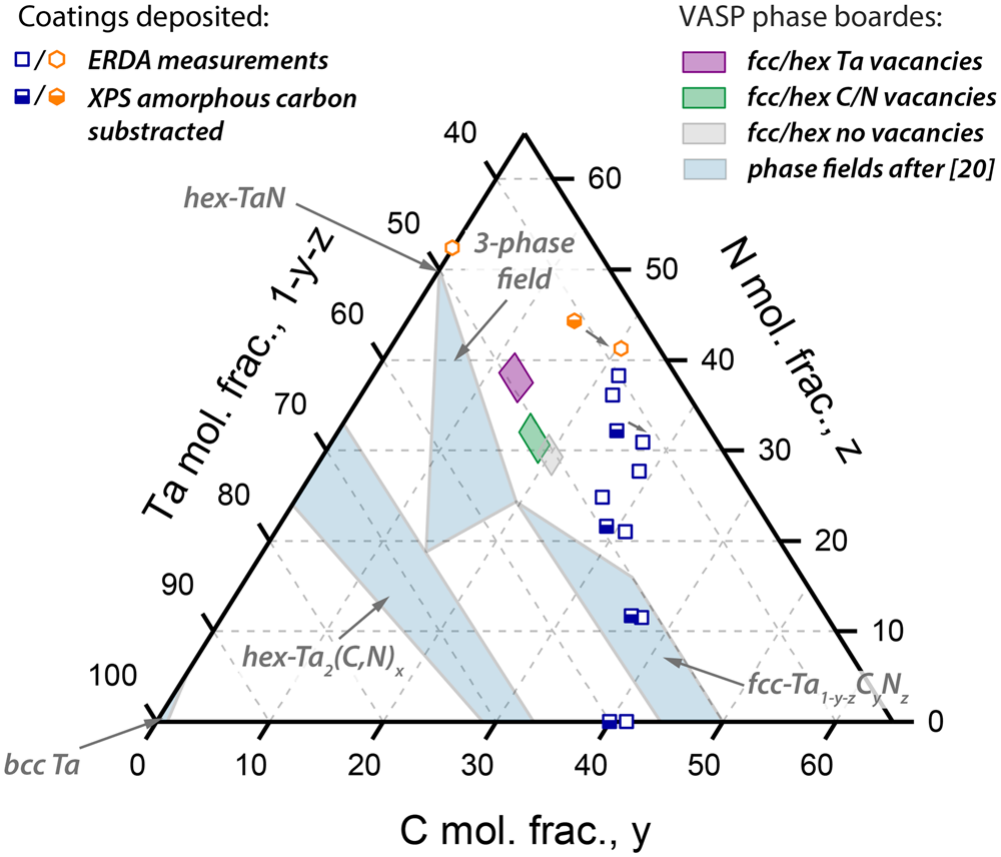
Ta_1−y−z_C_y_N_z_ ternary concentration triangle, with indicated phase fields reported by K. Frisk^20^ at 1400 °C. The ab initio obtained phase fields, see Fig. 2, are also indicated within the system. Open symbols represent compositions obtained by ERDA, whereas half-filled symbols are corrected by the amount of amorphous carbon based on XPS measurements.

The obtained structural and chemical data are plotted in a schematic Ta-C-N concentration triangle, which includes also some phase fields from isothermal section of the phase diagram calculated for 1400 °C^20^, Fig. 5. The single phase fcc coatings are presented by blue squares, whereas the mixed phased coatings (with minor fractions of hexagonal phases) are presented by orange hexagons. This presentation shows that actually only one ternary composition, Ta_0.51_C_0.37_N_0.12_, would be within the fcc- Ta_1−y−z_C_y_N_z_ single-phase field (according to equilibrium state conditions as used by ref.^20^). All other single-phase ternary fcc-Ta_1−y−z_C_y_N_z_ coatings are significantly outside this fcc- Ta_1−y−z_C_y_N_z_ single-phase field. This again confirms the strong impact of point defects and kinetic restrictions (as obtained by PVD) in stabilizing the fcc structure. As the formation of amorphous grain boundary phase fraction is typically a problem when preparing carbide coatings through reactive deposition, we estimated the amorphous free carbon by evaluating the C-C contributions in the C1s XPS spectra (not shown here). The peak fitting indicates that only 6% of all C atoms in the Ta_0.59_C_0.41_ film are present as non-Ta bonded carbon. The ternary Ta_0.51_C_0.37_N_0.12_ film exhibits an even lower amount of around 4%, whereas a further increase in the nitrogen content leads to more amorphous phase fractions up to 32% for Ta_0.38_C_0.20_N_0.42_ (please remember, we always used the same TaC compound target and added more or less N_2_ gas to prepare the ternary Ta_1−y−z_C_y_N_z_ coatings). These amorphous C-C contributions are subtracted from the ERDA measurements (open symbols in Fig. 5), leading to estimated C contents (bonded to Ta), which are presented by half-filled symbols. The overview (Fig. 5) also shows that the experimentally obtained transition between fcc and hex phases (at around x = 0.68) nicely coincides with the DFT obtained transition (purple, green, and grey shaded areas in Figs 2 and 5), especially when considering Ta vacancies and the corrected C content (subtracting the non-Ta-bonded contribution).

**Figure 6:**
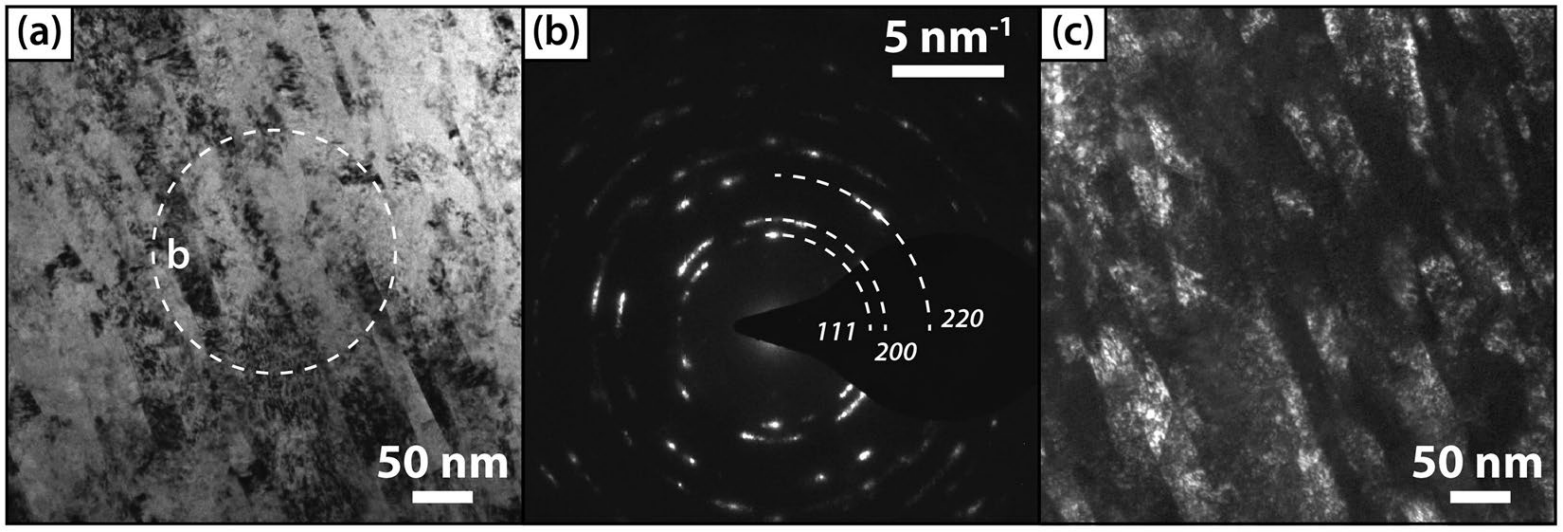
Cross sectional TEM bright field (BF) image (**a**), with corresponding SAED-pattern (**b**). A dark field (DF) image of Ta_0.46_C_0.32_N_0.22_ is presented in (**c**).

Detailed investigations of the microstructure of our coatings are conducted by cross-sectional SEM and TEM studies. In Fig. 6a a representative bright field image of Ta_0.46_C_0.32_N_0.22_ is given, exhibiting a highly dense and columnar growth morphology. The Selected Area Electron Diffraction (SAED) pattern plotted in Fig. 6b confirms the XRD measurements, indicating a polycrystalline fcc-structured thin film. In addition, dark field investigations reveal a columnar width of about 27 ± 5 nm for Ta_0.48_C_0.31_N_0.21_ compared to 14 nm obtained during X-ray diffraction of the powdered coatings. Further TEM investigations on different compositions also feature the trend of smaller column width with increasing nitrogen content. Pure Ta_0.59_C_0.41_ exhibits a larger column width of about 31 ± 6 nm. In addition, deposition rates were obtained out of the morphological investigations showing a clear decrease from 18.3 nm/min for Ar atmospheres, to 9 nm/min for ${{\rm{f}}}_{[{{\rm{N}}}_{2}]}^{{\rm{norm}}}$ = 0.5. Increasing the bias potentials also slightly decreases the deposition rates, independent on the nitrogen f  low rate ratio.

**Figure 7:**
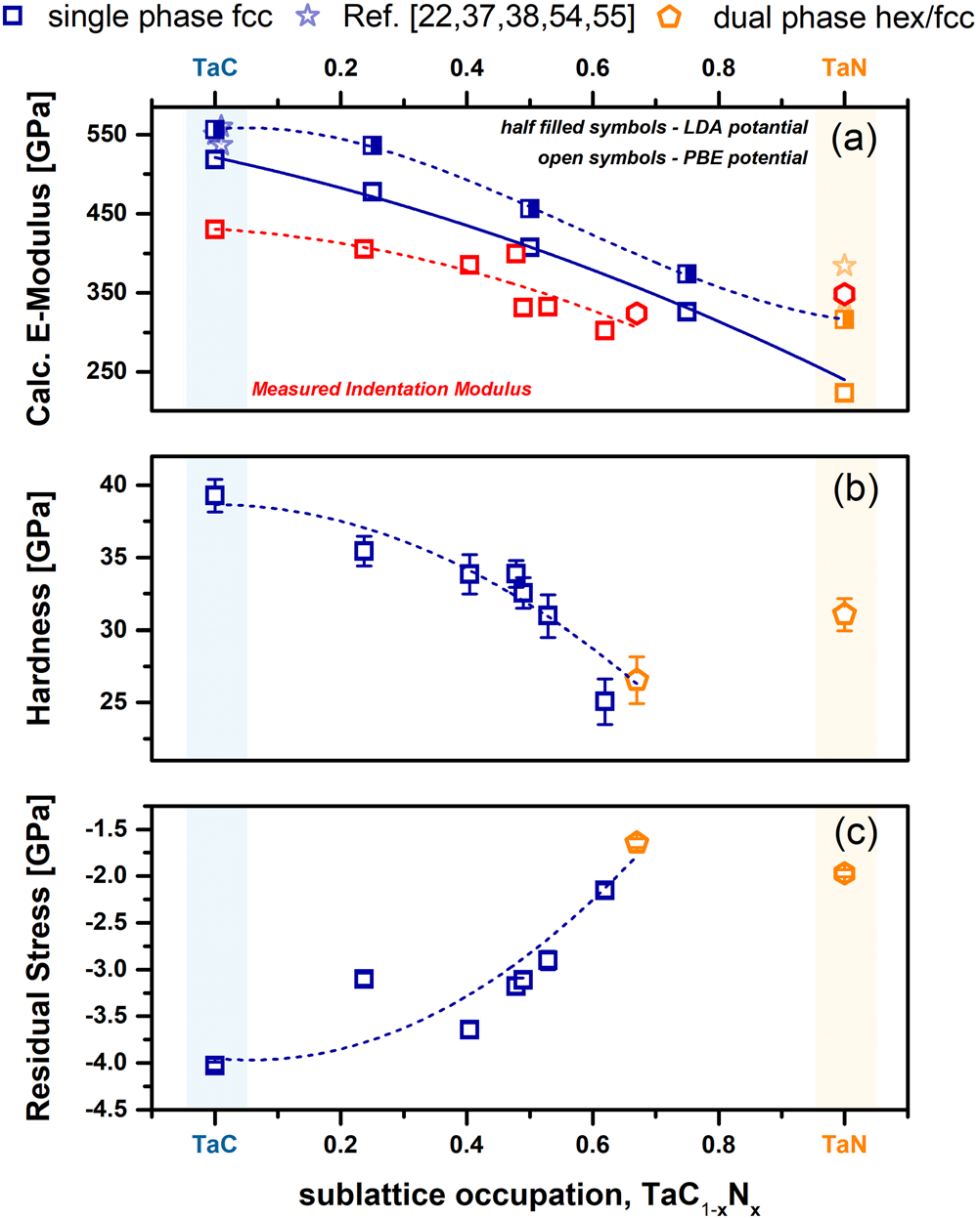
(**a**) VASP calculated E-modulus for fcc structured TaC_1−x_N_x_ utilizing different potentials, PBE and LDA, compared to literature and experimentally data (red symbols) determined by nanoindentation. Indentation hardness (**b**) as well as residual stress (**c**) in relation to the observed sublattice occupation. Hexagons indicate dual phased fcc/hex Ta(C,N) structures^22,37,38,54,55^.

As the main motivation of this work is to tailor the mechanical properties – especially enhancing ductility - of Ta-C thin films by substituting carbon through nitrogen, we also studied this by ab initio calculations. Elastic constants as well as polycrystalline bulk and shear modulus (B and G), Poisson ratio (ν), and Young’s modulus, E, applying Hill’s average^35^ of the Reuss and Voigt bulk moduli (B_R_ and B_V_) and shear moduli (G_R_ and G_V_), respectively, were calculated (Table 2). The detailed procedure of estimating B and G can be found in ref.^36^. The obtained results for the calculated Young’s modulus for fcc structured TaC_1−x_N_x_ – utilizing also different potentials – are presented in Fig. 7a. An increase of the nitrogen content clearly decreases the Young’s modulus from around 500 GPa for TaC to around 275 GPa for TaN, in relation to the used potentials. The results for the boarder systems fcc-Ta-C and fcc-Ta-N are in good agreement with values reported in literature (TaC^37^, fcc-TaN^22,38^). The slight deviations, especially for TaN, may also be related to the anisotropic behavior of this ceramic material. The low anisotropy factor of cubic TaC (0.6) and TaN (0.17) imply high anisotropy of the respective Young’s moduli. TaC_0.25_N_0.75_ exhibits an anisotropy factor closest to 1 (isotropy) among all material studied here, with 0.75 (LDA) and 0.76 (PBE). Thus, all materials are actually highly anisotropic, and a given texture will have a substantial influence on the mechanical properties of the films. Comparing now the theoretical predictions with experimentally observed indentation moduli – see red squares and hexagons in Fig. 7a – we can find a highly similar trend. All experimental values shown in Fig. 7 were measured on films deposited on silicon substrates with an applied bias voltage of −10 V. For comparison with the calculations the Ta-normalized notation is used here also for the experimental compositions. The general offset of about 50 GPa can be related to overestimating the mechanical properties in DFT by assuming defect-free crystals with no morphological features as well as no textural considerations. The decreased indentation modulus of TaC_0.71_, as it would be expected from the calculated slope, can be related to the non-stoichiometric composition of the Ta-C film, which lowers the elastic modulus^1^. Literature data for TaN are slightly higher as the measured and calculated values, being mostly related to textural effects, whereby the highest Young’s modulus should be obtained for the [100] direction. The deposited Ta-N contains small amounts of hexagonal TaN, which is however reported to have a higher Young’s modulus than fcc-TaN^39^ explaining the increase compared to Ta_0.38_C_0.20_N_0.42_.

**Figure 8:**
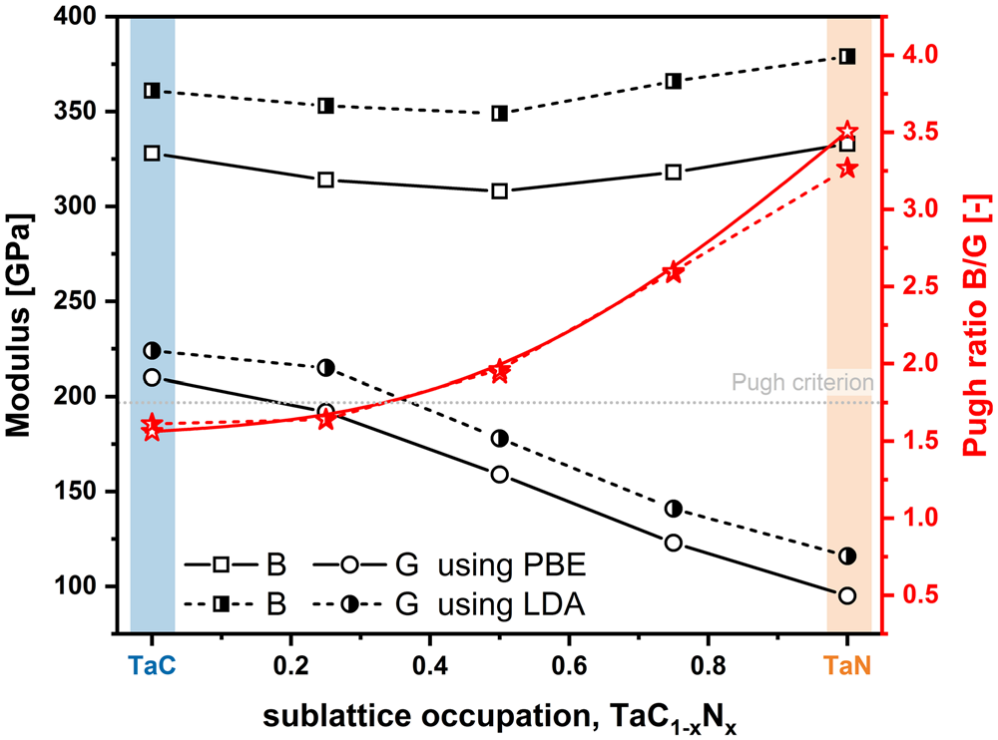
Polycrystalline bulk (B) and shear modulus (G) calculated with two different exchange correlation potentials – PBE and LDA. The empirical measure for ductility – Pugh’s ratio B/G – is plotted in red with open and half-filled stars for PBE and LDA potential respectively. B/G values above 1.75 are associated with the ductile character.

Next to the qualitative agreement between the calculated Young’s modulus and the experimentally measured indentation modulus, similar compositionally-driven trend is obtained also for the hardness. From almost superhard TaC_0.71_, 39.3 ± 1.0 GPa, the hardness monotonically decreases to around 26.4 ± 2.0 GPa for Ta_0.38_C_0.20_N_0.42_. Here, again the likely dual phase structure could slightly influence the mechanical properties, but still the decreasing trend is evident – indicating a more metallic like character. Furthermore, the substitution of carbon through nitrogen also decreases the highly compressed state of the films from −4.0 GPa for TaC_0.71_ to −1.75 GPa for Ta_0.38_C_0.20_N_0.42_. The difference in stress and hardness loss – over 10 GPa for the hardness compared to just 2.0 GPa for the residual stresses – is a further proof for the intrinsic property adjustment through alloying nitrogen on the non-metallic sublattice. In addition, increasing bias potentials (−90 V) increase the residual stress to around −4.63 GPa. Taking all this into account the experimentally determined and calculated trends of the mechanical behavior are in very good accordance. At this point, we have to mention that a different behavior is reported by S. Du *et al*.^24^. To estimate the brittle to ductile behavior of different material classes, semi empirical criteria have been employed. Commonly used criteria include the B/G ratio (Pugh-ratio)^40^ as well as Pettifor’s Couchy pressure (C_12_-C_44_)^41^. However, more recently, Niu *et al*. proposed a universal ductile to brittle criterion ((C_12_-C_44_)/E)^42^. Ductile behavior of materials is typically associated with B/G > 1.75, C_12_-C_44_ and C_12_-C_44_/E > 0. Therefore, we plotted B/G (red stars), B (black squares), and G (black circles) – as obtained by PBE and LDA potentially – as a function of the non-metal sublattice population x in Fig. 8. The solid lines (connecting the open symbols) refer to PBE and the dashed lines (connecting the half-filled symbols) refer to LDA potentials. Our calculations propose that the substitution of C atoms with N (for the investigated TaC_1−x_N_x_ supercells) leads to an increase in ductility according to each of the three criteria. In Fig. 8 this is only shown for the Pugh-criterion. When fitting the data points, the transition from brittle to ductile behavior is around x = 0.37 and 0.36 for LDA and PBE, respectively. For the other two criteria we obtain a transition around x = 0.34 for LDA and 0.35 for PBE potentials. Therefore, all three criteria are in excellent agreement. Cantilever bending tests confirm this prediction and are discussed in more detail in a follow-up study (K_IC_, of 2.9 ± 0.25 MPa·m^1/2^ for Ta_0.47_C_0.34_N_0.19_ (x = 0.36) compared to only 1.79 MPa·m^1/2^ for TaC_0.81_).

## Conclusion

Here, we studied how N alloying of fcc structured Ta-carbides, influences their structure, phase stability and mechanical properties. Ab initio predictions are corroborated by experimental studies of magnetron sputtered Ta-C-N thin films.

DFT calculations revealed, that the preferred vacancy type (metallic or non-metallic) of fcc Ta-C-N materials strongly depends on their non-metal composition (i.e., C or N content). Nitrogen rich compositions prefer metallic vacancies, whereas non-metallic vacancies are preferred for C rich compositions. Through this vacancy-stabilizing-effect of the fcc structure, the phase field for fcc Ta-C-N can be extended up to nitrogen contents of about 68% (on the non-metallic sublattice). This theoretical prediction is in excellent agreement with compositional and structural analysis of sputter deposited TaC_1−x_N_x_ thin films. The theoretical transition between fcc and hex phases is between x = 0.58 and 0.78, being in excellent agreement with our experiments, suggesting x = 0.68. The mechanical properties, namely the indentation modulus as well as the hardness, decrease with increasing N content, which is in good agreement with the DFT predictions. Specifically, indentation modulus from 430 to 324 GPa and hardness from 40 to 26 GPa for TaC_0.71_ to TaC_0.32_N_0.68_, respectively. The calculated Young’s modulus decreases nearly linearly from 500 GPa for TaC to 250 GPa for TaN, thus overestimating the experimental values. The decrease in hardness is also accompanied by a decrease in compressive residual stresses from around −4.0 GPa (TaC_0.71_) to −1.5 GPa (TaC_0.32_N_0.68_). But as this decrease is significantly lower as that in hardness, the hardness reduction is not just based on a decrease in compressive stresses but rather on intrinsic changes towards a more metallic like bonding state. Furthermore, applying the Pugh and Pettifor-criteria, we found that increasing nitrogen content increases the ductile character of Ta-C-N coatings.

This study emphasizes an alternative alloying concept for ceramic-like thin film materials, by forming solid solutions on the non-metallic sublattice. The investigated Ta-C-N system gives a promising prospective for further ultra-high temperature ceramics with enhanced damage tolerance.

## Methods

Ta-C and Ta-C-N thin films were deposited using an UHV magnetron sputtering system (AJA Orion 5), utilizing a 3-inch TaC_0.97_ compound target (Plansee Composite Materials GmbH)^28^. Ta-N was deposited using just two 2-inch Ta targets. All Ta-C films were sputtered in Ar atmosphere (purity 99.999%) whereas Ta-C-N and Ta-N films were deposited in Ar and N_2_ gas mixtures. The Ta-C target was operated in pulsed mode (pulse frequency 150 kHz with a pulse width of 2576 ns), whereas the Ta targets were driven under DC current. All depositions were carried out at a working gas pressure of 0.4 Pa with a total gas flow rate of 20 sccm. By varying the nitrogen to total flow rate, the amount of N within the Ta-C-N coating was adapted $-{{\rm{f}}}_{[{{\rm{N}}}_{2}]}^{{\rm{norm}}}=\frac{{{\rm{f}}}_{[{{\rm{N}}}_{2}]}}{{{\rm{f}}}_{[{{\rm{N}}}_{2}]}+{{\rm{f}}}_{[{\rm{Ar}}]}}$ varied between 0.05 to 0.5 – for further details see Table 1. During the depositions, the heater temperature was set to 600 °C (corresponding to a substrate temperature of 425 ± 10 °C) combined with a bias voltage of −10 V. Prior to the deposition processes, all targets and substrates were sputter-cleaned in Ar atmospheres (total pressure of 6.0 Pa). All coatings were deposited on polished single crystalline Si strips ((100) oriented, 20 × 7 × 0.38 mm³), as well as burnished single crystalline Al_2_O_3_ platelets ((1–102) oriented, 10 × 10 × 0.5 mm³). Furthermore, selected compositions were additionally deposited on 0.05-mm-thin low-alloy steel foils, which were subsequently dissolved in nitric acid, to obtain substrate-free coating materials. These were mechanically ground (by hand) with a ceramic mortar to receive coating powders.

**Table 1:** Chemical composition of the coatings obtained by ERDA analysis with the corresponding deposition parameters. The average error ranges between 1 and 2% of the presented mean atomic fractions.

Sample	Deposition parameters				N/(C+N)	Elements (mean atomic fraction)		
	Bias potential	${{\bf{f}}}_{[{{\bf{N}}}_{{\bf{2}}}]}^{{\bf{norm}}}$	Ar	N_2_	x	Ta	C	N
	[V]		[sccm]					
Ta_0.59_C_0.41_	−10	0	20	0	—	0.585	0.415	—
Ta_0.51_C_0.37_N_0.12_	−10	0.05	19	1	0.24	0.514	0.371	0.115
Ta_0.47_C_0.34_N_0.19_^a^	−75	0.05	19	1	0.36	0.466	0.344	0.190
Ta_0.48_C_0.31_N_0.21_	−10	0.1	18	2	0.40	0.481	0.309	0.210
Ta_0.46_C_0.32_N_0.22_^b^	−60	0.1	18	2	0.41	0.459	0.320	0.221
Ta_0.48_C_0.27_N_0.25_	−10	0.15	17	3	0.48	0.482	0.270	0.248
Ta_0.43_C_0.29_N_0.28_	−10	0.2	16	4	0.49	0.435	0.288	0.276
Ta_0.42_C_0.27_N_0.31_	−10	0.3	14	6	0.53	0.416	0.275	0.309
Ta_0.42_C_0.22_N_0.36_	−10	0.4	12	8	0.62	0.417	0.222	0.361
Ta_0.38_C_0.20_N_0.42_	−10	0.5	10	10	0.67	0.383	0.203	0.414
Ta_0.48_N_0.52_	−10	0.5	10	10	—	0.476	—	0.524

^a^Film was deposited at a heater temperature of 730 °C and power density of 0.75 W/cm^2^.

^b^Different bias potential was applied to study its effect on thin film morphology and hardness.

**Table 2:** DFT calculations using two different exchange correlation potential approximations, PBE and LDA.

	x	a_c_	C_11_	C_12_	C_44_	G	B	E	ν	B/G
PBE	0	4.482	702	139	172	210	328	518	0.24	1.56
	0.25	4.474	658	141	157	192	314	478	0.25	1.64
	0.5	4.462	585	169	133	159	308	407	0.28	1.94
	0.75	4.443	513	222	110	123	318	327	0.33	2.59
	1	4.423	672	162	42	95	333	260	0.37	3.51
LDA	0	4.421	768	157	181	224	361	556	0.24	1.61
	0.25	4.409	769	144	167	215	353	536	0.25	1.64
	0.5	4.395	684	183	141	178	349	456	0.28	1.96
	0.75	4.375	589	254	125	141	366	374	0.33	2.61
	1	4.356	792	173	52	116	379	316	0.36	3.26

Table 2 shows lattice constant (a_c_ in Å), elastic constants of stiffness tensor (C_11_, C_12_, C_44_), G, B, and Young’s moduli (all in GPa), Poisson’s ratio (ν) and Pugh’s ratio (B/G) in relation to the non-metallic alloying content, x, for fcc structured TaC_1−x_N_x_.

Structural investigations of all coatings (either on substrates as well as substrate-free powders) were done by X-Ray diffraction (XRD) in Bragg-Brentano configuration applying a Panalytical Empyrean diffractometer equipped with a Cu-K_α_ radiation source (wave length λ = 1.54 Å). The chemical composition of all thin films was investigated by Time-of-Flight Elastic Recoil Detection Analysis (TOF-ERDA)^43^ with a 36 MeV I^8+^ primary ion beam under a recoil detection angle of 45°. In order to avoid surface contaminations, the chemical compositions were evaluated for depths below at least 30 nm. The expected systematic uncertainties for the light elements (C and N) in the Ta-C-N system are found to be at most 5 to 10% of the detected concentrations for absolute measurements free from standards. This error is mainly due to uncertainties in the specific energy loss of the recoiling particles. Much higher accuracy is obtained for inter-sample comparison, as it is done within this study. Specific sources and consequences of systematic uncertainties are discussed in more detail by Zhang *et al*.^44^. In addition, to access the chemical bonding states within the films, we utilized X-ray Photoelectron Spectroscopy (XPS) – using a Physical Electronics Quantera II with monochromatic Al-K_α_ radiation and a take-off angle of 45° for the photoelectrons. High-resolution spectra were acquired after ion-etching (45 min with 200 eV Ar^+^ ions) the films on Si (100)-substrates. The analysis area was 100 µm in diameter and the ion-etched area about 1 × 1 mm². The coating morphology as well as structural evolution – especially with respect to nitrogen content – was investigated by electron microscopy utilizing a scanning electron microscope (SEM, FEI Quanta 250 FEGSEM operated at 5 keV) as well as a transmission electron microscope (TEM FEI TECNAI G20, acceleration voltage of 200 kV).

Indentation modulus, E, and hardness, H, were obtained by nanoindentation experiments carried out on coated Si as well as sapphire substrates utilizing an UMIS – equipped with a Berkovich diamond tip. At least 25 load displacement curves (per sample) were recorded at different loads (varying between 3 to 45 mN), which were analyzed after Oliver and Pharr^45^. Residual stresses within the thin films were obtained through the modified Stoney equation^46^ and evaluating the curvature of coated Si substrates by an optical profilometer (Nanovea PS50).

To investigate and describe the transition between fcc and hex structured TaC_1−x_N_x_ solid solutions, we utilized Density Functional Theory (DFT) calculations performed by the VASP (Vienna ab initio simulation package) code^47^^,^ using the projector-augmented plane-wave (PAW) pseudopotentials^48^. To gain a deeper insight on the effect of a chosen exchange-correlation (xc) potential, we compared two different standard approximations namely, Local Density Approximation (LDA)^49^ and Perdew-Burke-Ernzerhof Generalized Gradient Approximation (GGA-PBE)^50^. We used the following well-known crystallographic structures of the binaries: fcc, Fm$\bar{3}\,$m, #SG 225, and hex, P$\bar{6}$2m, #SG 189. From the conventional 8-atomic (fcc) and 6-atomic (hex) unit cells, 2 × 2 × 2 and 2 × 2 × 3 supercells with 64 and 72 atoms, respectively, were created. Various TaC_1−x_N_x_ compositions are obtained by substituting carbon atoms with nitrogen utilizing the Special Quasi-random Structure (SQS) method^51^. The influence of structural defects was investigated by a systematic removal of either metallic or non-metallic species, also applying SQS. The Brillouin zone of the cubic supercell was sampled with 6 × 6 × 6 k-points, the hexagonal supercell with 5 × 5 × 6 k-points and a plane wave cut-off energy of 600 eV was chosen based on performed convergence test with the aim to guarantee total energy accuracy of about 1 meV/at. Equilibrium lattice parameters and ground state energies were obtained by relaxing the supercell volumes, shapes and atomic positions (ISIF = 3 tag in VASP). Elastic constants of the TaC_1−x_N_x_ materials were calculated using the Universal Linear-Independent Coupling Strain (ULICS) method^52^.

## Data Availability

The datasets generated and analyzed during the current study are available from the corresponding author on reasonable request.

## References

[1] Riedl H (2018). Influence of carbon deficiency on phase formation and thermal stability of super-hard TaC y thin films. Scr. Mater..

[2] Yate L, Coy LE, Aperador W (2017). Robust tribo-mechanical and hot corrosion resistance of ultra-refractory Ta-Hf-C ternary alloy films. Sci. Rep..

[3] Williams WS (1999). Electrical properties of hard materials. Int. J. Refract. Met. Hard Mater..

[4] Pierson, H. O. *Handbook of refractory carbides and nitrides: properties, characteristics, processing, and applications*. (Noyes Publications, 1996).

[5] Mayrhofer PH (2003). Self-organized nanostructures in the Ti-Al-N system. Appl. Phys. Lett..

[6] Cedillos-Barraza O (2016). Investigating the highest melting temperature materials: A laser melting study of the TaC-HfC system. Sci. Rep..

[7] Malinovskis P (2018). Synthesis and characterization of multicomponent (CrNbTaTiW)C films for increased hardness and corrosion resistance. Mater. Des..

[8] Fahrenholtz, W. G., Wuchina, E. J., Lee, W. E. & Zhou, Y. Ultra-High Temperature Ceramics: Materials for ExtremeEnvironment Applications.

[9] Evans AG, Cannon RM (1986). Overview no. 48: Toughening of brittle solids by martensitic transformations. Acta Metall..

[10] Karch J, Birringer R, Gleiter H (1987). Ceramics ductile at low temperature. Nature.

[11] Zhang S, Wang HL, Ong S-E, Sun D, Bui XL (2007). Hard yet Tough Nanocomposite Coatings – Present Status and Future Trends. Plasma Process. Polym..

[12] Bermejo, R. *et al*. Hierarchical Architectures to Enhance Structural and Functional Properties of Brittle Materials. *Adv. Eng. Mater*. **19** (2017).

[13] Hahn R (2016). Superlattice effect for enhanced fracture toughness of hard coatings. Scr. Mater..

[14] Moraes V (2018). Ab initio inspired design of ternary boride thin films. Sci. Rep..

[15] Sangiovanni DG, Chirita V, Hultman L (2010). Electronic mechanism for toughness enhancement in Ti × M 1 − x N (M = Mo and W). Phys. Rev. B.

[16] Edström D (2018). Elastic properties and plastic deformation of TiC- and VC-based pseudobinary alloys. Acta Mater..

[17] Kindlund H (2013). Toughness enhancement in hard ceramic thin films by alloy design. APL Mater..

[18] Kindlund H (2014). Vacancy-induced toughening in hard single-crystal V0.5Mo0.5Nx/MgO(0 0 1) thin films. Acta Mater..

[19] Grumski M, Dholabhai PP, Adams JB (2013). Ab initio study of the stable phases of 1:1 tantalum nitride. Acta Mater..

[20] Frisk K (1998). Analysis of the phase diagram and thermochemistry in the Ta–N and the Ta-C-N systems. J. Alloys Compd..

[21] Pacher F, Mayrhofer PH, Holec D (2017). Vacancy-driven extended stability of cubic metastable Ta-Al-N and Nb-Al-N phases. Surf. Coatings Technol..

[22] Koutná, N., Holec, D., Friák, M., Mayrhofer, P. H. & Šob, M. Stability and elasticity of metastable solid solutions and superlattices in the MoN-TaN system: a first-principles study. 1–12 (2017).

[23] Yu X, Thompson GB, Weinberger CR (2015). Influence of carbon vacancy formation on the elastic constants and hardening mechanisms in transition metal carbides. J. Eur. Ceram. Soc..

[24] Du S (2017). N dependent tribochemistry: Achieving superhard wear-resistant low-friction TaC × N y films. Surf. Coatings Technol..

[25] Chang KS (2010). Physical and chemical characterization of combinatorial metal gate electrode Ta-C-N library film. Appl. Phys. Lett..

[26] Aouadi SM (2006). Physical and chemical properties of sputter-deposited TaC × N y films. J. Phys. Condens. Matter.

[27] Koutná N, Holec D, Svoboda O, Klimashin FF, Mayrhofer PH (2016). Point defects stabilise cubic Mo-N and Ta-N. J. Phys. D. Appl. Phys..

[28] Lasfargues H (2017). Non-reactively sputtered ultra-high temperature Hf-C and Ta-C coatings. Surf. Coatings Technol.

[29] Tasnádi F, Lugovskoy AV, Odén M, Abrikosov IA (2017). Non-equilibrium vacancy formation energies in metastable alloys — A case study of Ti0.5Al0.5N. Mater. Des.

[30] Bohr N (1913). I. On the constitution of atoms and molecules. London, Edinburgh, Dublin Philos. Mag. J. Sci.

[31] Powder diffraction file 00-035-0801. International Center for Diffraction Data. *PDF-4*+ (2015).

[32] Powder diffraction file 00-049-1283. International Center for Diffraction Data. *PDF-4*+ (2015).

[33] Powder diffraction file 03-065-9404. International Center for DiffractionData. *PDF-2* (2007).

[34] Powder diffraction file 04-010-9382. International Center for Diffraction Data. *PDF-4*+ (2015).

[35] Hill R (1952). The elastic behaviour of a crystalline aggregate. Proc. Phys. Soc. Sect. A.

[36] Tasnádi F, Odén M, Abrikosov IA (2012). Ab initio elastic tensor of cubic Ti 0.5Al 0.5N alloys: Dependence of elastic constants on size and shape of the supercell model and their convergence. Phys. Rev. B - Condens. Matter Mater. Phys..

[37] López-De-La-Torre L, Winkler B, Schreuer J, Knorr K, Avalos-Borja M (2005). Elastic properties of tantalum carbide (TaC). Solid State Commun..

[38] Chang J, Zhao G-P, Zhou X-L, Liu K, Lu L-Y (2012). Structure and mechanical properties of tantalum mononitride under high pressure: A first-principles study. J. Appl. Phys..

[39] Zhao E, Hong BO, Meng J, Wu Z (2009). First principles investigation on the ultra-incompressible and Hard TaN. J. Comput. Chem..

[40] Pugh SF (1954). XCII. Relations between the elastic moduli and the plastic properties of polycrystalline pure metals. London, Edinburgh, Dublin Philos. Mag. J. Sci..

[41] Pettifor DG (1992). Theoretical predictions of structure and related properties of intermetallics. Mater. Sci. Technol..

[42] Niu, H. *et al*. Extra-electron induced covalent strengthening and generalization of intrinsic ductile-to-brittle criterion. *Scientific Reports* **2**, 718, 10.1038/srep00718 (2012). 10.1038/srep00718 PMC3466921 23056910

[43] Ström P, Petersson P, Rubel M, Possnert G (2016). A combined segmented anode gas ionization chamber and time-of-flight detector for heavy ion elastic recoil detection analysis. Rev. Sci. Instrum..

[44] Zhang Y (1999). Detection efficiency of time-of-flight energy elastic recoil detection analysis systems. Nucl. Instruments Methods Phys. Res. Sect. B Beam Interact. with Mater. Atoms.

[45] Oliver WC, Pharr GM (1992). An improved technique for determining hardness and elastic modulus using load and displacement sensing indentation experiments. J. Mater. Res..

[46] Janssen GCAM, Abdalla MM, van Keulen F, Pujada BR, van Venrooy B (2009). Celebrating the 100th anniversary of the Stoney equation for film stress: Developments from polycrystalline steel strips to single crystal silicon wafers. Thin Solid Films.

[47] Kresse G, Furthmüller J (1996). Efficient iterative schemes for *ab initio* total-energy calculations using a plane-wave basis set. Phys. Rev. B.

[48] Kresse G, Joubert D (1999). From ultrasoft pseudopotentials to the projector augmented-wave method. Phys. Rev. B.

[49] Kohn W, Sham LJ (1965). Self-Consistent Equations Including Exchange and Correlation Effects. Phys. Rev..

[50] Perdew JP, Burke K, Ernzerhof M (1996). Generalized Gradient Approximation Made Simple. Phys. Rev. Lett..

[51] Wei S-H, Ferreira LG, Bernard JE, Zunger A (1990). Electronic properties of random alloys: Special quasirandom structures. Phys. Rev. B.

[52] Yu R, Zhu J, Ye HQ (2010). Calculations of single-crystal elastic constants made simple. Comput. Phys. Commun..

[53] Powder diffraction file 04-008-1849. International Center for Diffraction Data. *PDF-4*+ (2015).

[54] Jun CK, Shaffer PTB (1971). Elastic moduli of niobium carbide and tantalum carbide at high temperature. J. Less Common Met..

[55] Brown HL, Armstrong PE, Kempter CP (1966). Elastic Properties of Some Polycrystalline Transition-Metal Monocarbides. J. Chem. Phys..

